# Construction and clinical validation of a machine learning-based risk prediction model for poor wound healing after anorectal surgery

**DOI:** 10.3389/fsurg.2026.1829528

**Published:** 2026-09-09

**Authors:** Liao Pan, Lizhi He, Jianzhen Zhao, Keya Li, Juan Gao, Zhenquan Wang

**Affiliations:** Proctology Center, The Second Affiliated Hospital of Hunan University of Chinese Medicine, Changsha, Hunan, China

**Keywords:** anorectal surgery, clinical validation, machine learning, random forest, risk prediction, wound healing

## Abstract

**Objective:**

To construct and validate a machine learning-based risk prediction model for poor wound healing after anorectal surgery, enabling early identification and individualized intervention.

**Methods:**

Clinical data from 246 patients (Jan 2022–Dec 2024) were retrospectively collected. Wound healing status at 4 weeks post-surgery defined outcome (good: *n* = 191; poor: *n* = 55). Independent predictors were identified via multivariate Logistic stepwise regression. Patients were split 7:3 into training and internal validation sets. Three models—Logistic regression, gradient boosting machine (GBM), and random forest (RF)—were developed. Performance was assessed via AUC, calibration curves, Brier scores, and decision curve analysis. Temporal validation was performed using 152 patients from the same center (Jan 2025–Dec 2025) as an independent time-based cohort.

**Results:**

BMI, diabetes, stool consistency, and preoperative perianal infection were common core predictors. In internal validation, AUCs were 0.893 (Logistic), 0.897 (RF), and 0.877 (GBM). In temporal validation, AUCs were 0.874 (Logistic), 0.865 (GBM), and 0.853 (RF). Logistic regression showed the smallest AUC fluctuation across training, internal, and temporal sets (0.864→0.893→0.874) and lowest Brier scores (0.092–0.123), indicating best calibration and generalizability. GBM achieved the highest training AUC (0.925) but significant performance decay in validation (*Δ*AUC = 0.048), suggesting overfitting. After grid search with 10-fold cross-validation, RF attained internal AUC of 0.897 and sensitivity of 0.800, close to Logistic regression, but temporal performance (AUC=0.853, Brier=0.129) was slightly inferior.

**Conclusion:**

Logistic regression demonstrated the best overall discriminative ability, calibration, clinical net benefit, and cross-cohort generalizability, and is recommended as the preferred tool for individualized risk assessment. RF, after thorough optimization, shows promise as an alternative. GBM was limited by sample size and exhibited overfitting, warranting further validation in larger studies. This study provides a quantitative framework for early risk stratification in anorectal surgery patients.

## Introduction

Anorectal diseases refer to a class of disorders occurring in the anal and rectal regions, mainly including anal fistula, anal fissure, perianal abscess, and hemorrhoids. Their typical clinical manifestations include constipation, diarrhea, hematochezia, and other symptoms, which seriously affect patients' quality of life (1). In recent years, with changes in lifestyle and dietary structure, the incidence of anorectal diseases has shown an increasing trend year by year, and they have become one of the most common digestive system diseases in clinical practice (2). Currently, a comprehensive regimen combining medication and surgery is commonly used in clinical practice to treat anorectal diseases. Medications can only provide short-term symptom relief, and long-term use may easily lead to gastrointestinal adverse reactions. Although anorectal surgery can completely remove the lesions, due to the special anatomical limitations of the anal region, postoperative wounds are mostly open wounds (3, 4). Patients are required to have normal bowel movements after surgery, and repeated stimulation of the wound can easily induce complications such as infection and bleeding, thereby leading to delayed wound healing (5, 6). Poor postoperative wound healing not only exacerbates patients' pain and prolongs the recovery period but also adds to the overall healthcare burden, making it an urgent clinical problem in anorectal surgery (7). At present, there is a lack of widely accepted quantitative risk stratification tools for poor wound healing after anorectal surgery in clinical practice. Clinical decision-making is mostly based on surgeons' assessment of known risk factors, and existing evaluation tools, including simple scoring systems and traditional nomograms, are difficult to fully capture the complex interactions among multiple factors (8, 9). Therefore, a more comprehensive and quantitative approach that integrates multiple risk factors and captures their complex interactions is needed to improve preoperative risk stratification.

In recent years, machine learning (ML) techniques have been increasingly widely applied in the medical field due to their unique advantages in processing high-dimensional data and mining nonlinear relationships (10, 11). Multiple studies have confirmed that ML algorithms such as random forest (RF), and gradient boosting machine (GBM) have demonstrated superior performance over traditional Logistic regression in predicting postoperative complications in colorectal surgery, cardiac surgery, orthopedics, and other fields (12–14). However, the comparative performance of these algorithms for predicting poor wound healing after anorectal surgery has not been systematically evaluated. Based on this, the present study aims to compare the performance of three machine learning algorithms—Logistic regression, gradient boosting machine, and random forest—in predicting poor wound healing after anorectal surgery, to screen for key predictive factors and validate the optimal model, thereby providing an objective and quantitative individualized risk assessment tool for clinical practice.

## Materials and methods

### Materials

A total of 246 patients who underwent surgical treatment in the Department of Anorectal Surgery in our hospital from January 2022 to December 2024 were selected, and their relevant medical records were collected for retrospective analysis. Inclusion criteria: (1) diagnosed with one of the following anorectal diseases prior to surgery: anal fistula, anal fissure, perianal abscess, or hemorrhoids (15); (2) complete medical records; (3) acceptance of anorectal surgical treatment; (4) number of incisions ≤3; (5) completion of one-stage radical surgery. Exclusion criteria: (1) patients with malignant tumors; (2) patients with coagulation dysfunction; (3) patients with mental disorders; (4) patients with previous history of anorectal surgery.

### Sample size and grouping

In this study, a total of 19 variables were included in the data compilation. The sample size should be 5–10 times the number of variables (16), thus requiring 95–190 cases. However, considering a 10%–12% rate of missing data, and after discussion with chief physicians with extensive statistical analysis experience in the Department of Anorectal Surgery, the relevant data of 246 patients who underwent anorectal surgery from January 2022 to December 2024 were finally selected. Based on the wound healing status at 4 weeks post-surgery, the patients were divided into a good healing group and a poor healing group, among which 55 cases (22.36%) had poor wound healing. The final model included 4 predictor variables, with an Events Per Variable (EPV) ratio of 55/4 = 13.75, which meets the minimum statistical requirement of EPV ≥10 (7).

Wound healing was assessed as follows (17): evaluated at 4 weeks postoperatively. Good healing: (1) disappearance of clinical symptoms: main symptoms such as pain and swelling completely disappeared; (2) good growth of granulation tissue: granulation tissue appeared as bright red granules with vigorous growth and no obvious exudate; (3) complete epithelialization of wound: wound covered with newly formed epithelium, dressing showed no obvious infiltration and no need for dressing change. Poor healing: 1. Persistent pain: postoperative pain was severe, affecting sleep and daily life, requiring analgesics. 2. Slow or absent growth of granulation tissue: granulation tissue appeared dull in color, grew slowly or not at all, with poor wound flatness. 3. Excessive exudate: wound exudate was abundant, soaking more than one-third of the dressing. 4. Prolonged healing time: wound healing time was significantly prolonged, exceeding the expected duration.

### Data collection

Based on literature review and expert panel knowledge, the following 15 factors were identified as important indicators that may affect the occurrence of poor wound healing in patients after anorectal surgery: patient age, sex, body mass index (BMI), diabetes mellitus, smoking history, disease type, incision selection, mechanical stimulation, stool consistency (Bristol Stool Form Scale, types 3–5 considered good consistency, types 1–2 and 6–7 considered poor consistency (18), preoperative perianal infection (defined as infection present at the time of surgery, including perianal abscess and anal fistula, which are infectious conditions by nature), malnutrition (albumin <35 g/L), operation time, surgical procedure, etc. The diagnosis of perianal infection was established preoperatively based on clinical findings (localized erythema, swelling, tenderness, and purulent discharge) and intraoperative confirmation.

### Construction and evaluation of machine learning algorithm prediction models

R software (Version 4.0.3) was used to randomly select 70% of the data as the training set, and the remaining 30% as the internal validation set. The training set was used to construct the Logistic regression [using the Akaike Information Criterion (AIC) with forward stepwise selection to identify the optimal equation], gradient boosting machine (GBM), and random forest (RF) models, while the internal validation set was used to evaluate the predictive performance of the models. Accuracy, precision, recall, F1 score, area under the curve (AUC) value, and receiver operating characteristic (ROC) curve were used as evaluation metrics to assess the discriminative ability of the models. Calibration curves combined with the Hosmer-Lemeshow test were used to evaluate model calibration. Decision curve analysis (DCA) was used to evaluate the clinical net benefit of the models.

### Hyperparameter optimization

The hyperparameters of the gradient boosting machine and random forest were optimized within the training set using grid search combined with 10-fold cross-validation. The internal validation set was completely unseen during the tuning process to ensure an unbiased estimate of the model's generalization ability.

The search range for the gradient boosting machine (GBM) was as follows: learning rate (shrinkage): 0.05, 0.1, 0.2; tree depth (interaction.depth): 3, 4, 5; minimum sample size per leaf node (n.minobsinnode): 5, 10; number of base trees (n.trees): 500, 1000, 2,000. With the maximization of cross-validated AUC as the selection criterion, the final optimal parameter combination was determined as: learning rate = 0.05, tree depth = 4, minimum sample size per leaf node = 5, number of base trees = 500.

The search range for the random forest (RF) was as follows: number of candidate variables per node (mtry): 1, 2, 3, 4; minimum sample size per node (nodesize): 1, 5, 10; number of trees (ntree): 200, 500, 1,000, 2,000. The final optimal parameter combination was determined as: mtry = 1, nodesize = 5, ntree = 200.

Logistic regression employed stepwise regression with AIC as the criterion for variable selection, and did not involve hyperparameter optimization.

### Statistical analysis

SPSS 26.0 software was used for data collation and univariate analysis. Normally distributed measurement data were expressed as mean ± standard deviation (x̅ ± s) and analyzed using t-test; non-normally distributed measurement data were expressed as [M(P25, P75)] and compared between groups using Wilcoxon rank sum test. Count data were expressed as number (percentage) [n(%)], and analyzed using *χ*^2^ test or Fisher's exact probability method. Delong test was used to compare AUC among different prediction models. *P* < 0.05 was considered statistically significant. All statistical tests were two-sided.

## Results

### Comparison of general characteristics

Among the 246 patients who underwent anorectal surgery in this study, 191 were in the good wound healing group and 55 were in the poor wound healing group, with an incidence of poor wound healing of 22.36%. The proportions of constipation, preoperative perianal infection, and diabetes mellitus in the poor wound healing group were higher than those in the good wound healing group, and the BMI level in the poor wound healing group was lower than that in the good wound healing group, with statistically significant differences (*P* < 0.05). See Table 1.

**Table 1:** Comparison of general characteristics.

Clinical Characteristics	Good Healing Group (*n* = 191)	Poor Healing Group (*n* = 55)	t/*χ*^2^	P
Age (x̅ ± s, years)	51.35 ± 13.85	52.15 ± 14.28	0.372	0.710
Sex [n(%)]			0.357	0.550
Male	113 (59.16)	35 (63.64)		
Female	78 (40.84)	20 (36.36)		0.576
BMI (x̅ ± s, kg/m^2^)	23.22 ± 3.28	20.57 ± 3.01	5.373	<0.001
Disease type [n(%)]			0.445	0.931
Anal fistula	68 (35.60)	22 (40.00)		
Anal fissure	8 (4.19)	2 (3.64)		
Perianal abscess	34 (17.80)	10 (18.18)		
Hemorrhoids	81 (42.41)	21 (38.18)		
Chronic constipation [n(%)]	36 (18.85)	12 (21.82)	0.240	0.624
Diabetes mellitus [n(%)]	18 (9.42)	16 (29.09)	13.867	<0.001
Hypertension [n(%)]	42 (21.99)	14 (25.45)	0.292	0.589
Thyroid disease [n(%)]	21 (10.99)	8 (4.19)	0.518	0.472
Tumor [n(%)]	6 (3.14)	3 (5.45)		0.423
Gastroenteritis [n(%)]	25 (13.09)	6 (10.91)	0.184	0.668
Smoking history	48 (25.13)	16 (29.09)	0.348	0.555
Incision selection [n(%)]			0.254	0.614
Completely open incision	143 (74.87)	39 (70.91)		
Semi-open incision	48 (25.13)	16 (29.09)		
Mechanical irritation [n(%)]	67 (35.08)	23 (41.82)	0.836	0.361
Stool consistency [n(%)]			43.976	<0.001
Poor	30 (15.71)	33 (60.00)		
Good	161 (84.29)	22 (40.00)		
Preoperative perianal infection [n(%)]	10 (5.24)	16 (29.09)	25.71	<0.001
Operation time (x̅ ± s, min)	17.46 ± 5.83	17.38 ± 5.51	0.070	0.944
Surgical procedure [n(%)]			0.297	0.586
Traditional	128 (67.02)	39 (70.91)		
Minimally invasive	63 (32.98)	16 (29.09)		
Local hematoma [n(%)]			0.362	0.547
Yes	15 (7.85)	3 (5.45)		
No	176 (92.15)	52 (94.55)		

### Stepwise regression Variable selection

All 15 candidate clinical indicators were included in the complete modeling cohort (*n* = 246) for conditional forward stepwise regression. The stepwise regression selection results are shown in Table 2. The variables were sequentially entered in the following order: stool consistency, preoperative perianal infection, BMI, and comorbid diabetes mellitus, with no variables being eliminated throughout the process. The final model had an adjusted R^2^ = 0.361, with an overall *F*-test value of 35.595 (*P* < 0.001), indicating that all four indicators were independent influencing factors for poor wound healing after anorectal surgery.

**Table 2:** Stepwise regression variable selection results.

Model	Added Variable	Adjusted R2	Overall F	*P* value
Model 1	Stool consistency	0.182	54.621	<0.001
Model 2	preoperative perianal infection	0.257	41.336	<0.001
Model 3	BMI	0.342	38.772	<0.001
Model 4	Comorbid diabetes mellitus	0.361	35.595	<0.001

### Construction of different prediction models based on machine learning algorithms

**Figure 1:**
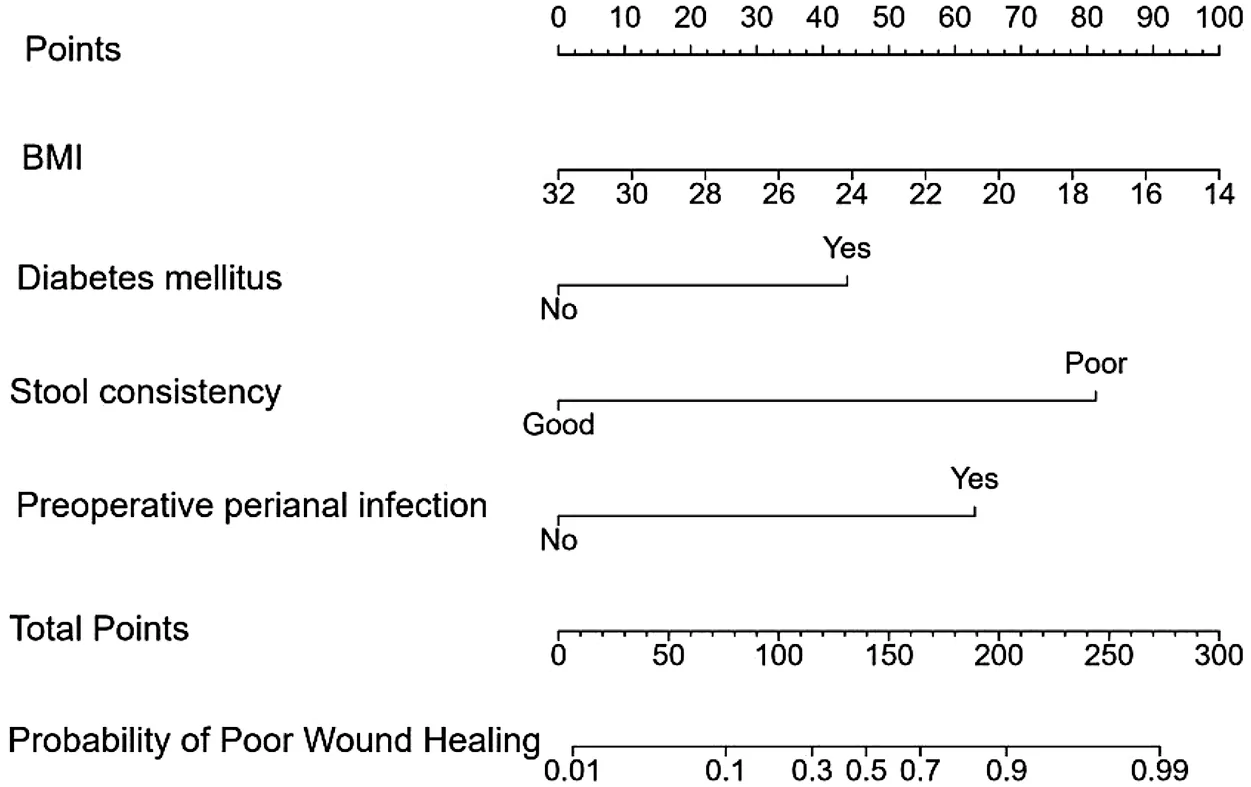
Visualized nomogram based on logistic regression model.

**Figure 2:**
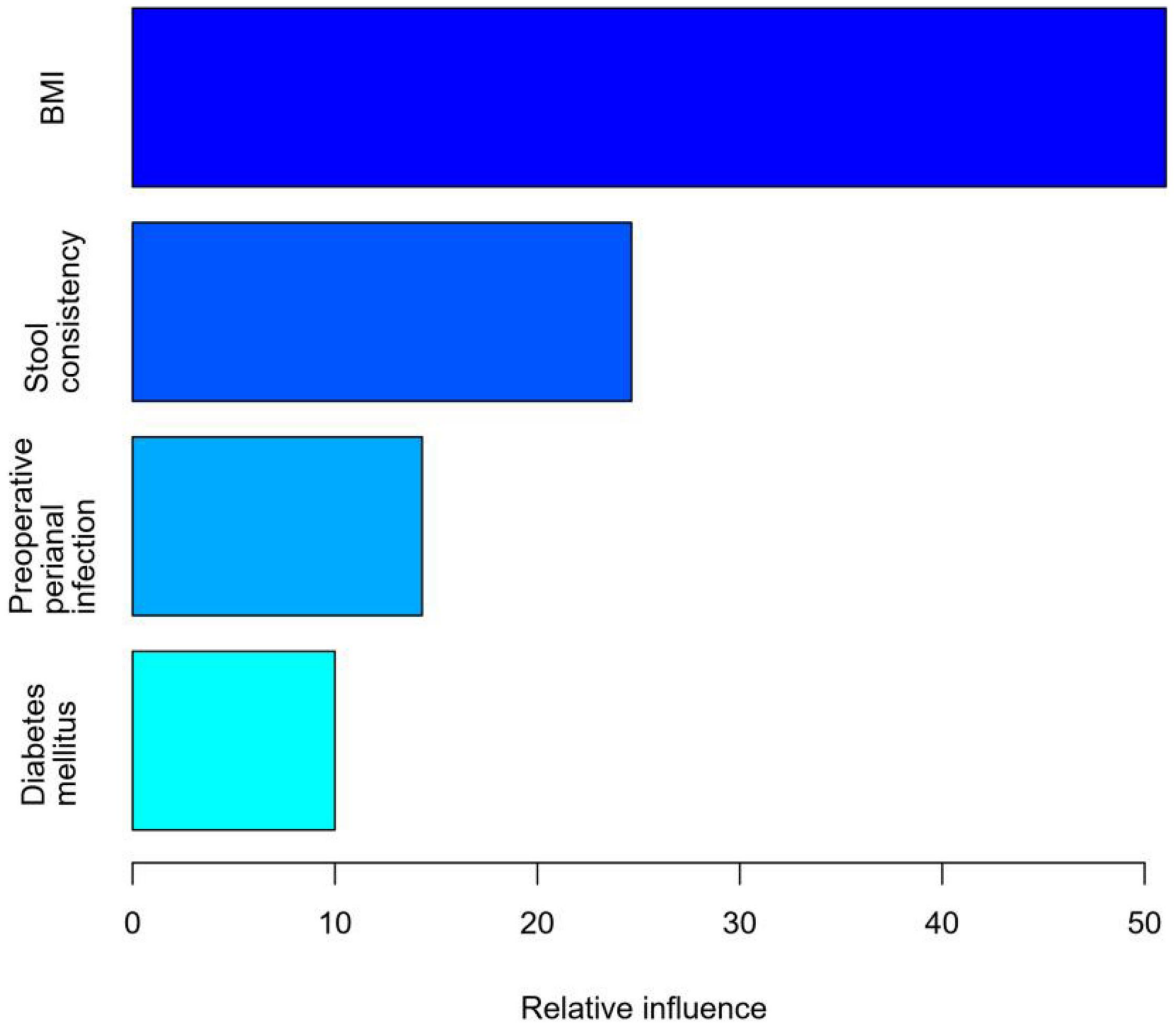
Gradient boosting machine model.

**Figure 3:**
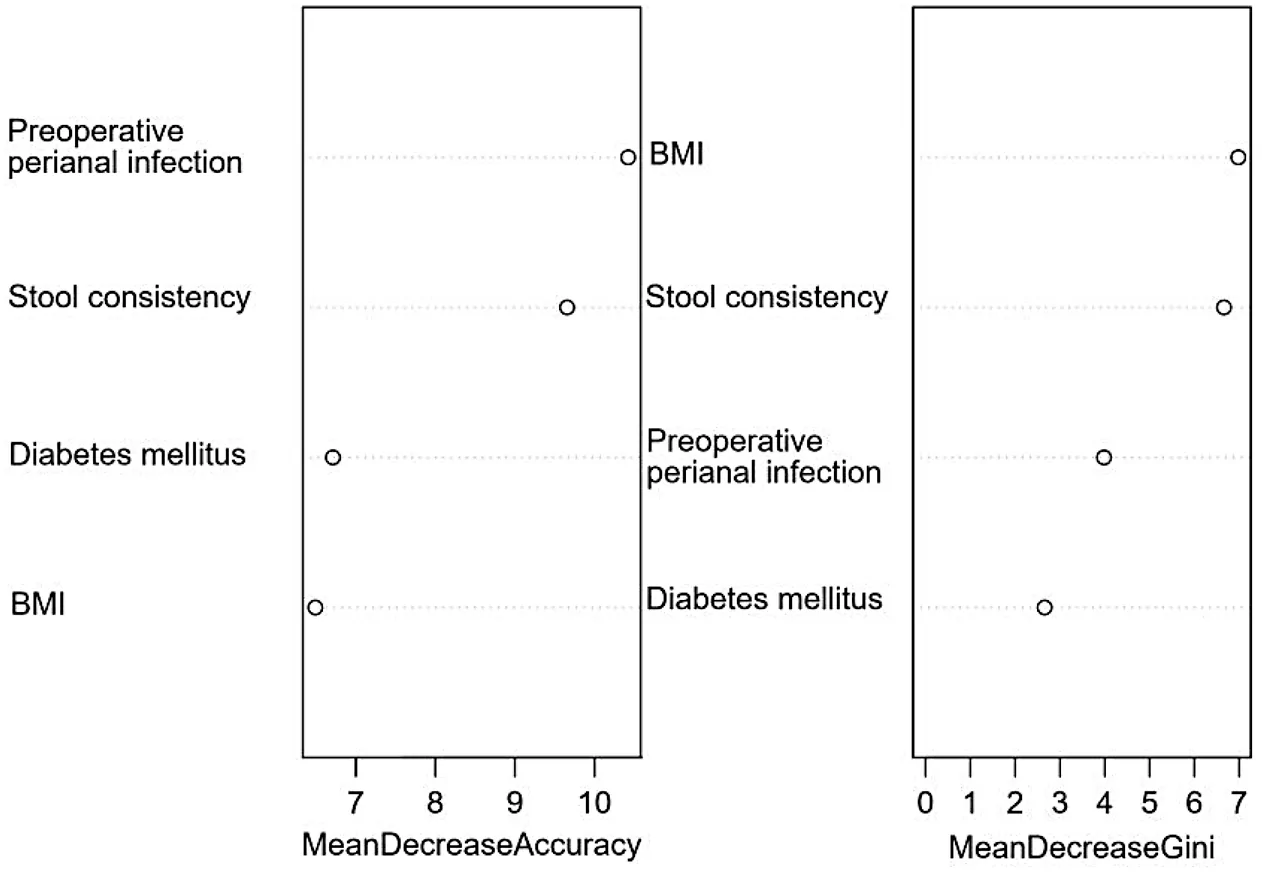
Random forest model.

R software (Version 4.0.3) was used to randomly select 70% of the data as the training set. With postoperative wound healing status after anorectal surgery as the dependent variable (good healing = 0, poor healing = 1), and the four independent predictors identified by multivariate Logistic stepwise regression as independent variables (variable assignments are shown in Table 3), the Logistic model (see Figure 1), gradient boosting machine model (see Figure 2), and random forest model (see Figure 3) were respectively constructed. The results of the Logistic regression model in the training set are shown in Table 4.

**Table 3:** Coding table.

Variable	Coding Method
BMI	Enter original value
Diabetes mellitus	Yes = 1, No = 0
Stool consistency	Poo*r* = 1, Good=0
Preoperative perianal infection	Yes=1, No = 0

**Table 4:** Results of the logistic regression model in the training set.

Variable	B	SE	Wald	P	OR (95% CI)
BMI	−0.192	0.079	5.873	0.015	0.826 (0.707–0.964)
Diabetes mellitus	1.506	0.599	6.331	0.012	4.510 (1.395–14.582)
Stool consistency	2.804	0.527	28.331	<0.001	16.510 (5.879–46.363)
Preoperative perianal infection	2.172	0.628	11.946	<0.001	8.777 (2.561–30.083)

### Evaluation results of prediction models with different machine learning algorithms

In the training set, the gradient boosting machine (GBM) demonstrated the best discriminative performance (AUC = 0.925, 95%CI 0.871–0.982), followed by Logistic regression (AUC=0.864, 95% CI 0.788–0.940), while random forest had the lowest AUC in the training set (0.827, 95%CI 0.752–0.914). All three models had the same sensitivity of 0.825 in the training set. Logistic regression and GBM had identical accuracy, specificity, precision, and F1 scores, whereas random forest had lower specificity and precision, indicating poorer overall fit compared to the other two models. The DeLong test results indicated that the AUC of GBM in the training set was significantly higher than that of random forest (*P* < 0.05), while the differences in AUC between GBM and Logistic regression, as well as between Logistic regression and random forest, were not statistically significant (*P* > 0.05).

**Figure 4:**
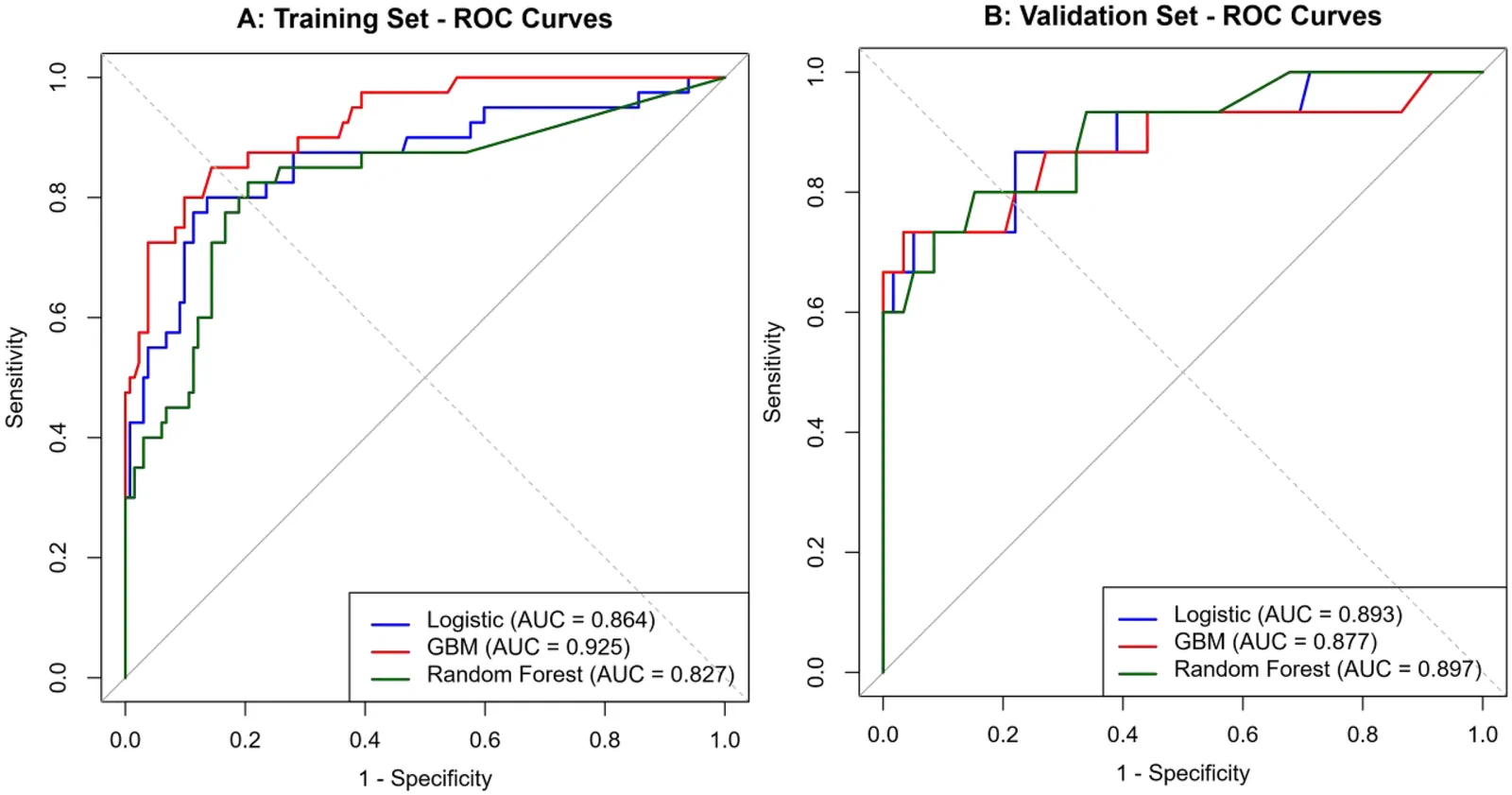
ROC curves of prediction models with different machine learning algorithms. **(A)** Training set; **(B)** Validation set.

**Figure 5:**
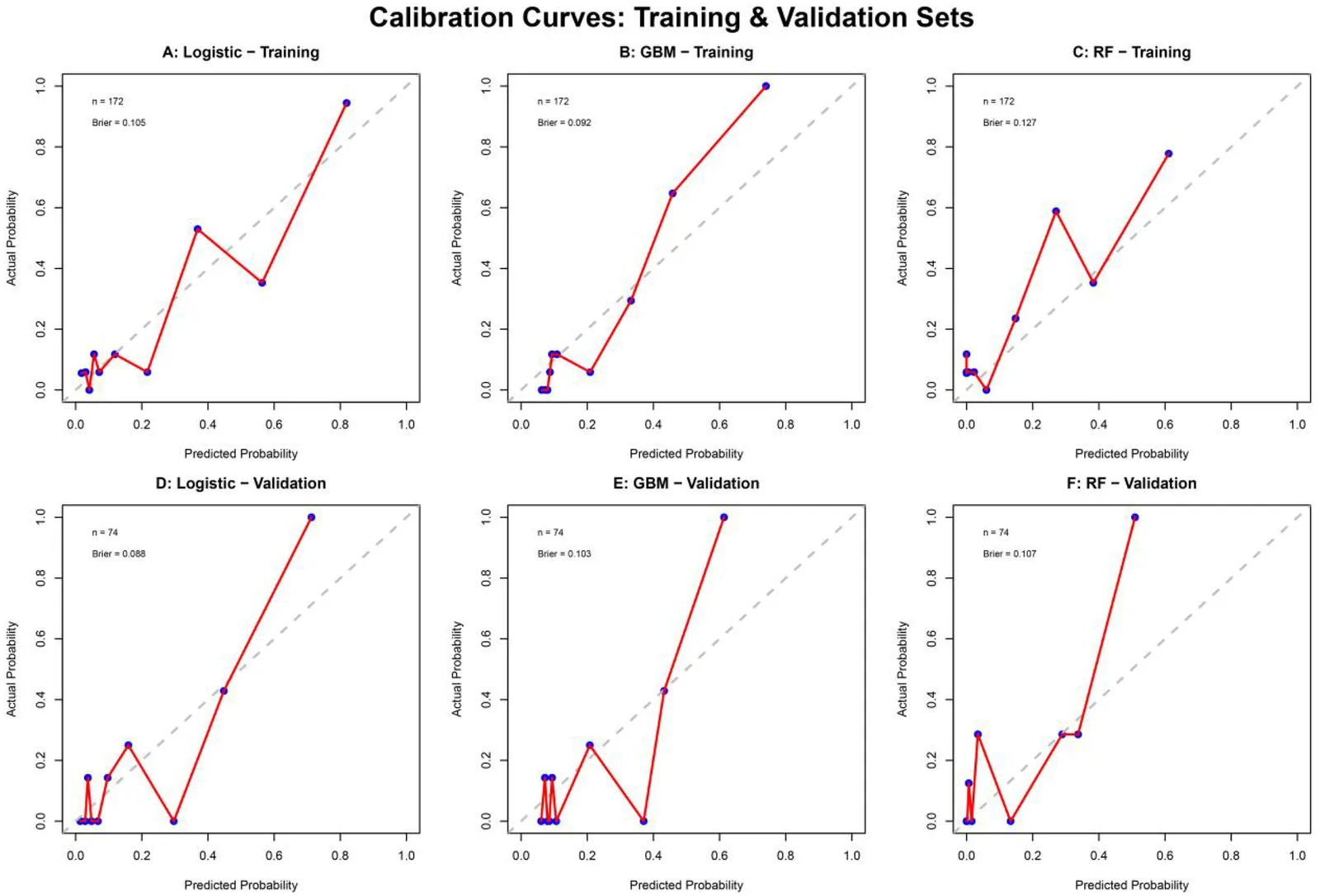
Calibration curves of the three models in the training set and internal validation set. **(A)** Logistic regression training set; **(B)** GBM training set; **(C)** RF training set; **(D)** Logistic regression internal validation set; **(E)**: GBM internal validation set; **(F)** RF internal validation set. The *x*-axis represents the model-predicted probability of poor wound healing, and the *y*-axis represents the actual probability of poor wound healing. The diagonal line represents the ideal perfect calibration reference line; the closer the curve is to the diagonal line, the higher the consistency between the model-predicted probability and the actual events. Each subplot is annotated with sample size **(n)** and Brier score; a lower Brier score indicates better calibration.

Upon switching to the internal validation set, the model performance showed clear divergence: Logistic regression had an AUC of 0.893(95% CI 0.787–0.999) and random forest had an AUC of 0.897(95%CI 0.786–0.996), with comparable discriminative abilities and identical accuracy, specificity, precision, and F1 scores. In contrast, the AUC of GBM in the validation set declined to 0.877 (*Δ*AUC = 0.048 from training), with simultaneous declines across all performance metrics, suggesting significant overfitting. At the optimal cutoff thresholds, all three models in the internal validation set had the same sensitivity of 0.800. Logistic regression demonstrated the best overall stability, and pairwise DeLong tests for AUC comparisons among the three models showed no statistically significant differences (*P* > 0.05) (Table 5, Figure 4, Figure 5).

**Table 5:** Evaluation results of prediction models based on different machine learning algorithms.

Model	Dataset	AUC	Accuracy	Sensitivity	Specificity	Precision	F1 Score
Logistic Regression	Training set	0.864	0.890	0.825	0.910	0.733	0.776
	Internal validation set	0.893	0.877	0.800	0.897	0.667	0.727
GBM	Training set	0.925	0.890	0.825	0.910	0.733	0.776
	Internal validation set	0.877	0.836	0.800	0.845	0.571	0.667
Random Forest	Training set	0.827	0.827	0.825	0.827	0.589	0.688
	Internal validation set	0.897	0.877	0.800	0.897	0.667	0.727

### Temporal validation

**Figure 6:**
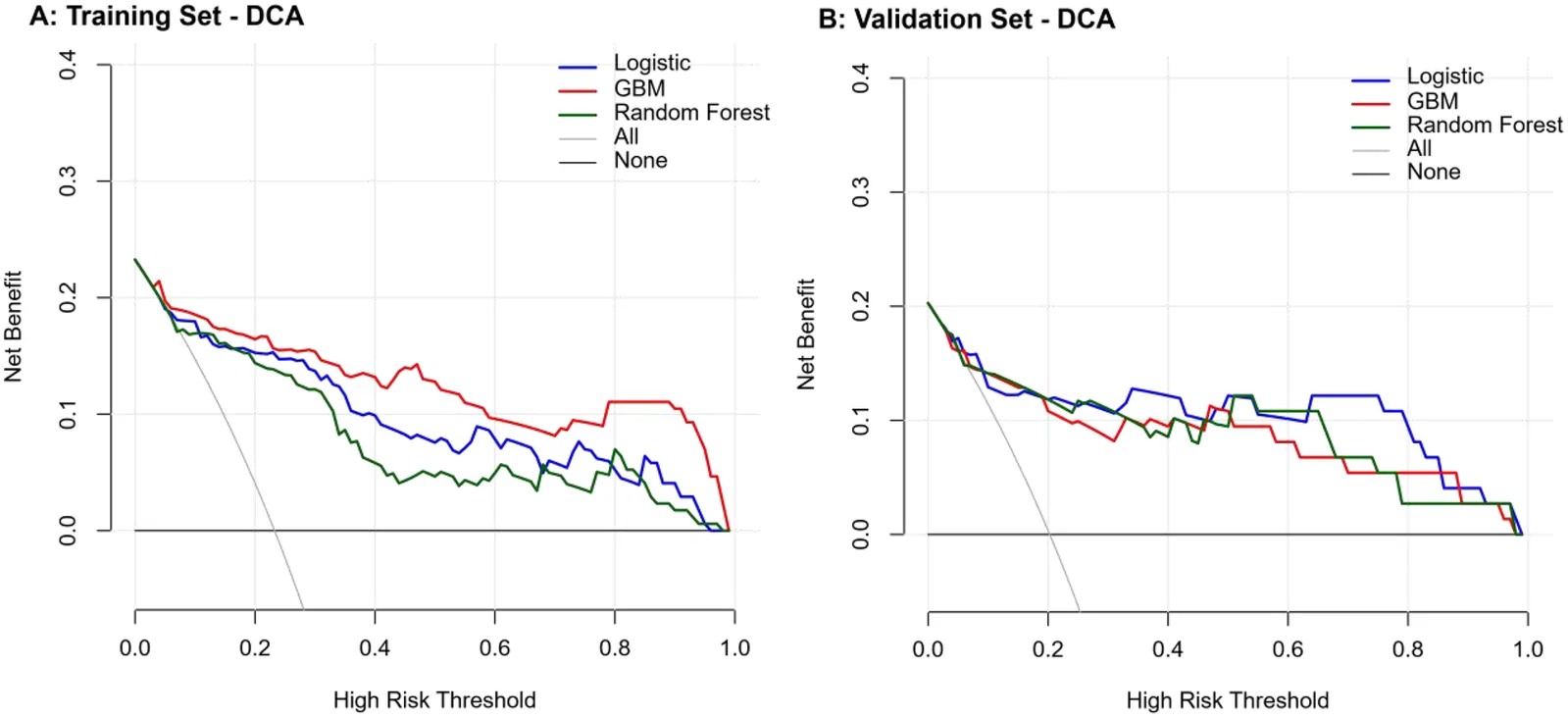
Training and internal DCA decision curves. (**A**) Training set; (**B**) Validation set.

**Figure 7:**
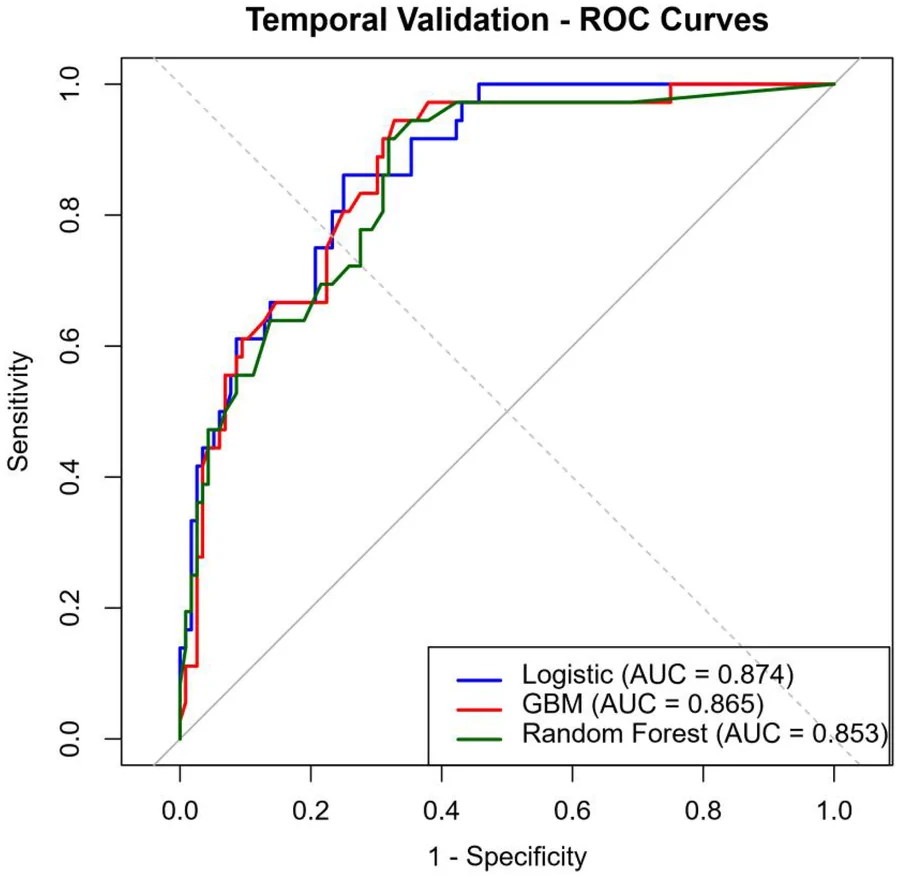
Temporal validation ROC curves.

**Figure 8:**
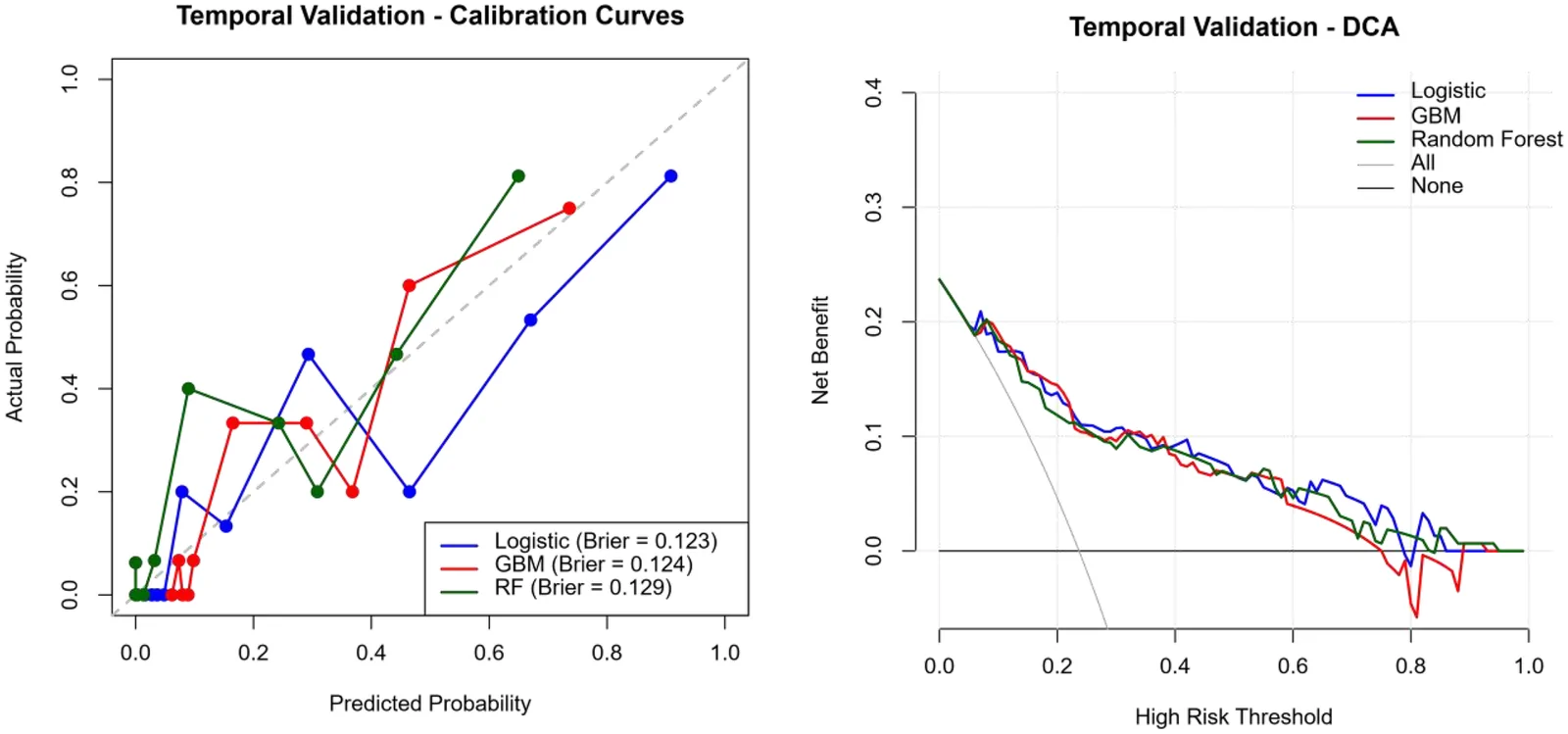
Temporal validation calibration curves and DCA decision curves.

A total of 152 cases were included in the temporal validation cohort, with 36 cases of poor wound healing, yielding an incidence rate of 23.68%. In the temporal validation cohort, Logistic regression achieved the highest AUC of 0.874 (95% CI 0.816–0.932), followed by GBM with an AUC of 0.865 (95% CI 0.802–0.928), and random forest with an AUC of 0.853 (95% CI 0.785–0.921). All three models had temporal AUC values > 0.85, indicating stable temporal discriminative ability. In terms of sensitivity, GBM (0.944) and random forest (0.917) outperformed Logistic regression (0.861), suggesting that machine learning algorithms are more suitable for large-scale population screening to reduce missed diagnoses among high-risk patients. However, Logistic regression had the best specificity of 0.750 and overall accuracy of 0.776, resulting in fewer misclassifications among low-risk individuals and more reliable individualized risk assessment. The Brier scores for each model in the temporal cohort were: Logistic = 0.123, GBM = 0.124, Random Forest = 0.129 (see Figure 6 Temporal Validation Calibration Curves). All three models had Brier scores < 0.25, indicating that calibration met clinically acceptable standards overall. The DCA results for the temporal cohort showed that within the commonly used clinical risk threshold range of 0.1–0.6, all three models had net benefits higher than the two reference lines of “treat all” and “treat none.” Logistic regression consistently demonstrated the highest net benefit across the entire threshold range (Table 6, Figures 7, 8).

**Table 6:** Predictive performance of the three models in the temporal validation cohort.

Model	AUC	Accuracy	Sensitivity	Specificity	Precision	F1 Score
Logistic Regression	0.874	0.776	0.861	0.750	0.517	0.646
GBM	0.865	0.737	0.944	0.672	0.472	0.630
Random Forest	0.853	0.737	0.917	0.681	0.471	0.623

## Discussion

Anorectal diseases are mostly caused by the special anatomical structure of the anorectum, improper diet, prolonged sedentary work, and other factors. These diseases not only present with complex and variable clinical symptoms but also significantly affect patients' daily work and quality of life (19). Surgery remains the primary curative approach for a considerable proportion of these patients. However, due to the invasive nature of the procedure and the special location of the lesions, postoperative wounds after anal fistula surgery are often left open, with large wound areas and slow healing (20). Without effective postoperative management, patients are prone to a series of problems including pain, poor wound healing, and recurrence of anal fistula, which seriously affect postoperative recovery (21, 22). Interventions to reduce the occurrence of poor postoperative wound healing include comprehensive preoperative assessment, refinement of intraoperative techniques, and early postoperative monitoring with targeted interventions (23). For example, poor preoperative glycemic control has been confirmed as an important risk factor affecting postoperative wound healing and should be actively corrected before surgery (24, 25). For patients with high-risk factors such as diabetes mellitus and obesity, intraoperative procedures should be as precise as possible to shorten operative time and reduce secondary damage to wound tissue; postoperative care should emphasize wound management, infection control, and optimization of bowel movement management to promote healing. However, the above measures are mostly empirical interventions and lack precise risk stratification for individual patients. Therefore, individualized preoperative risk assessment and implementation of corresponding preventive measures based on the assessment results are key to reducing the incidence of poor wound healing after anorectal surgery.

In this study, the three modeling algorithms uniformly identified four independent predictive factors: BMI, stool consistency, preoperative perianal infection, and comorbid diabetes mellitus. The mechanisms by which each indicator affects wound healing are consistent with existing clinical research findings: ① BMI: BMI is an intuitive indicator of the body's nutritional reserve and is highly correlated with the wound repair process (26, 27). In clinical practice, surgeons often focus on optimizing surgical techniques and may overlook preoperative nutritional status screening. Nutritional assessment should be incorporated into routine preoperative evaluation, with enhanced support provided for malnourished patients. ② Poor stool consistency: Hard and dry stools increase abdominal pressure during defecation, repeatedly pull on the fresh surgical wound, induce local edema and microvascular rupture with bleeding, inhibit granulation tissue epithelialization, and significantly delay wound healing (28). ③ Comorbid diabetes mellitus: Hyperglycemia inhibits vascular endothelial cell proliferation, causing microcirculatory hypoxia at the wound site and impaired protein synthesis, ultimately resulting in chronic non-healing wounds. It is a well-established independent risk factor for poor postoperative wound healing (29–31); postoperative wounds after anorectal surgery are chronically exposed to fecal contamination, and inflammation is more difficult to control in diabetic patients, further exacerbating wound damage. ④ Preoperative perianal infection: Persistent fecal contamination of the wound induces sustained inflammatory infiltration, directly prolonging the wound healing cycle. Qiao et al. (28) compared clinical data between patients with good healing and poor healing, confirming that preoperative perianal infection is an independent risk factor for poor prognosis.

Compared with traditional Logistic regression, machine learning offers three incremental advantages: first, it does not require pre-specification of interaction terms and can automatically mine nonlinear synergistic effects among multiple variables; second, it can output variable importance rankings to intuitively quantify the overall predictive contribution of each indicator; third, it has good data compatibility and can integrate multimodal data such as imaging and time-series laboratory results (8, 9). In this study, the training set AUC of GBM was significantly higher than that of Logistic regression (0.925 vs. 0.864), suggesting that machine learning may have greater potential for performance improvement under large sample conditions. However, the realization of machine learning advantages depends on a sufficient number of positive event samples. In this study, there were only 55 cases of poor wound healing, with an events per variable (EPV) ratio of 13.75. Although this met the minimum modeling standard, high-capacity models such as GBM and RF are prone to overfitting random noise in the training set, resulting in inflated training performance, performance decay in independent cohorts, and overestimation of adverse events in medium-risk ranges (11). Based on the combined internal and temporal validation results, the Logistic regression model demonstrated the best overall stability for predicting poor wound healing after anorectal surgery. GBM only showed outstanding discriminative ability in the training set (AUC = 0.925), with notable performance decay upon entering the internal (AUC = 0.877, *Δ*AUC = 0.048) and temporal validation cohorts (AUC = 0.865), indicating overfitting; random forest had an internal validation AUC close to that of Logistic regression, but its discriminative ability and calibration performance declined in the temporal cohort. Logistic regression had the smallest AUC fluctuation across the training, internal, and temporal cohorts, with stable cross-population discriminative ability. In terms of calibration performance, Logistic regression maintained Brier scores in the range of 0.092–0.123 across all cohorts, which were lower than those of GBM and random forest. Its calibration curve closely followed the ideal diagonal line, indicating better alignment between predicted probabilities and actual adverse event rates. Based on DCA decision curve analysis, within the commonly used clinical risk threshold range of 0.1–0.6, Logistic regression consistently had higher net clinical benefit than GBM, random forest, and the two reference curves of “treat all” and “treat none.” When the risk threshold exceeded 0.6, the net benefit of GBM dropped to negative values, indicating that guiding interventions based on this model at high cutoffs provided no clinical benefit, whereas Logistic regression maintained stable clinical application value throughout. A risk probability of ≥0.10 is recommended as the cutoff threshold for initiating enhanced preoperative interventions.

However, this study has several limitations. First, although temporal validation was performed using an independent cohort from a later time period, both the development and validation cohorts originated from a single center, which may limit generalizability to broader populations. Second, the poor healing outcome occurred in only 55 patients. Although the EPV ratio (55/4 = 13.75) met the minimum statistical requirement (≥10) for Logistic regression, this limited sample size may still be insufficient for complex ensemble algorithms such as GBM and random forest, potentially contributing to the observed overfitting and calibration drift. Third, our analysis was restricted to preoperative static clinical variables, without incorporating postoperative dynamic indicators such as serial C-reactive protein trajectories or pain score changes. Future multicenter prospective studies with larger sample sizes are needed to validate these findings, and the integration of time-series data through dynamic modeling approaches may further improve predictive performance and facilitate real-time risk updating during the postoperative period.

## Data Availability

The original contributions presented in the study are included in the article/Supplementary Material, further inquiries can be directed to the corresponding author.
